# Parental developmental experience affects vocal learning in offspring

**DOI:** 10.1038/s41598-024-64520-8

**Published:** 2024-06-14

**Authors:** Fanny-Linn H. Kraft, Ondi L. Crino, Saidat O. Adeniran-Obey, Raven A. Moraney, David F. Clayton, Julia M. George, Katherine L. Buchanan

**Affiliations:** 1 School of Life and Environmental Sciences, Deakin University, Geelong, Australia; 2 Department of Zoology, Stockholm University, Stockholm, Sweden; 3 College of Science and Engineering, Flinders University, Bedford Park, SA Australia; 4 School of Biological and Chemical Sciences, Queen Mary University of London, London, UK; 5 Department of Genetics and Biochemistry, Clemson University, Clemson, SC USA; 6 Department of Biological Sciences, Clemson University, Clemson, SC USA

**Keywords:** Birdsong, Developmental stress, Gene expression, Intergenerational effects, Transgenerational plasticity, Zebra finch, Zoology, Animal behaviour, Neuroscience, Cognitive neuroscience, Epigenetics in the nervous system, Gene expression

## Abstract

Cultural and genetic inheritance combine to enable rapid changes in trait expression, but their relative importance in determining trait expression across generations is not clear. Birdsong is a socially learned cognitive trait that is subject to both cultural and genetic inheritance, as well as being affected by early developmental conditions. We sought to test whether early-life conditions in one generation can affect song acquisition in the next generation. We exposed one generation (F1) of nestlings to elevated corticosterone (CORT) levels, allowed them to breed freely as adults, and quantified their son’s (F2) ability to copy the song of their social father. We also quantified the neurogenetic response to song playback through immediate early gene (IEG) expression in the auditory forebrain. F2 males with only one corticosterone-treated parent copied their social father’s song less accurately than males with two control parents. Expression of ARC in caudomedial nidopallium (NCM) correlated with father-son song similarity, and patterns of expression levels of several IEGs in caudomedial mesopallium (CMM) in response to father song playback differed between control F2 sons and those with a CORT-treated father only. This is the first study to demonstrate that developmental conditions can affect social learning and neurogenetic responses in a subsequent generation.

## Introduction

Environmental conditions can cause heritable variation in phenotypic traits without changes in genetic sequence^1^. In recent years, such trans- and intergenerational plasticity has attracted considerable attention due to the potential to allow for rapid evolutionary change^2,3^, but to date, there are few convincing examples of rapid, adaptive change^3^. Furthermore, such rapid phenotypic change can be the product of multiple complex mechanisms through both genetic and cultural inheritance (i.e., social transmission of behavioural phenotypes), which both influence trait expression but through different mechanisms^4,5^. Socially learned traits may be particularly susceptible to such intergenerational effects, as these traits may be influenced by epigenetic as well as cultural inheritance^6–8^. While there has been growing interest in transgenerational plasticity and debate regarding its adaptive potential, few studies have examined the transgenerational impact of environmental change on culturally inherited traits.

Bird song represents an excellent trait with which to test how the environment can cause phenotypic change across generations, as this trait is determined by both genetic and cultural inheritance^4,9^. Indeed, there is some evidence for more rapid evolutionary change in species that show learned rather than innate song structures^9^. Songbirds acquire their songs through a social learning process that is analogous to human speech development^10–12^, and this learning process is controlled by well-documented neural mechanisms and restricted to specific developmental windows^13^. During early development, juvenile songbirds acquire a song template from a tutor during a sensitive developmental window and use this template to form a version of the species’ song^14^. Adverse early-life conditions can impact vocal learning through detrimental effects on the development of the underlying neurological mechanisms^15–17^ and motor skills^18–20^, although it is worth noting that there can be beneficial impacts of elevated glucocorticoid levels^21,22^. Such developmental impacts are thought to be mediated by repeated acute or sustained chronic impacts on corticosterone (CORT) levels, the principle avian glucocorticoid produced in response to environmental challenges^23^. Glucocorticoids are secreted from the adrenal gland in response to activation of the hypothalamic–pituitary–adrenal (HPA) axis, a cascade that is triggered by neuronal signals sent from hierarchical neural systems to the hypothalamus. These hormones play a number of key physiological roles in optimising long-term fitness primarily to balance metabolic demands^24^, which are particularly crucial during growth and development, as compensatory growth may not be possible after the stressor has been removed without fitness costs^25^. Indeed, CORT is implicated in mediating the effect on both song production (e.g. singing rate,^19^) and song copy accuracy (i.e. song learning,^26^), due to impacts on the neural development associated with song production^15,27^. Song production affects mate choice and other social interactions, and variation in song structure can impact individual fitness as well as population dynamics ^9,28,29^. Song learning in birds, therefore, represents an excellent system for examining how the developmental environment can affect the expression of a fitness-related cognitive trait at both the behavioural and neural levels. However, to date, studies have not addressed whether early developmental conditions affect the intergenerational cultural inheritance mechanisms of songs by altering song-learning accuracy and/or the saliency of tutor song.

Vocal learning has been assessed functionally by quantifying the accuracy of song copying or, at the mechanistic level, using neural expression of immediate early genes (hereafter IEGs,^30^). These genes are differentially expressed in neural centres associated with song acquisition, including the caudomedial nidopallium (NCM) and mesopallium (CMM), in response to song playback. The magnitude of this response depends on the salience^31–33^ and novelty^34,35^ of the stimulus. For example, playback of conspecific song and tutor song has been shown to result in elevated EGR1 expression in the NCM in zebra finches (*Taeniopygia castanotis*,^36,37^), but the same gene showed lower expression to a song after habituation^35,38^, indicating that salience, as well as novelty, affects its expression. Male zebra finches exposed to early-life food restriction showed reduced IEG expression following tutor song playback compared to control siblings, indicating that IEG expression reflects the effects of developmental conditions on song learning^39^. Furthermore, males exposed to early-life food restriction had reduced neuronal activation to song playback, showing that the electrophysiological response mirrored IEG expression levels^39^. This study indicated that the long-term impacts of developmental conditions on song learning are, in part, due to alterations in the formation of auditory memories. As song is a trait that is transmitted through cultural inheritance, it is possible that developmental conditions could affect song learning across generations, but the intergenerational impact of developmental conditions on vocal learning remains untested. The existence of such intergenerational effects could have profound implications for both social interactions and population dynamics, for example, through dialect formation and mate choice, which affect population-level processes^40,41^.

The aim of the current study was to test whether early-life conditions in one generation can have cascading consequences for auditory memory formation and song production in a subsequent generation. To address this, we experimentally manipulated early developmental conditions in a generation of zebra finches using CORT. We then observed whether there were consequences in the next generation on the production of, or IEG reactivity to, learned song. First, we predicted that parental treatment would affect offspring song copying, such that individuals with at least one CORT-treated parent would show reduced tutor copy accuracy. Second, we predicted that expression of key genes known to be upregulated in response to recognition of tutor songs in the NCM or CMM following tutor song playback would be affected by maternal treatment, paternal treatment, or an interaction of both treatments^30^. Third, in line with published studies testing within-generation impacts, we predicted that the similarity between the song playback and the male’s own song would be correlated with IEG expression^33,42^. To the best of our knowledge, this represents the first experimental test of the effects of early developmental conditions on vocal learning across generations.

## Results

### Treatment effects on song characteristics

We found no evidence of an effect of the treatment on any of the song characteristics (Weiner entropy, fundamental frequency, mean frequency) or measures of song complexity number of syllables, song length) in either F1 or F2 males, indicating that the treatment had no strong effect on song structure (P > 0.2 for all tested variables).

### Parental treatment affects song copying by offspring

**Figure 1:**
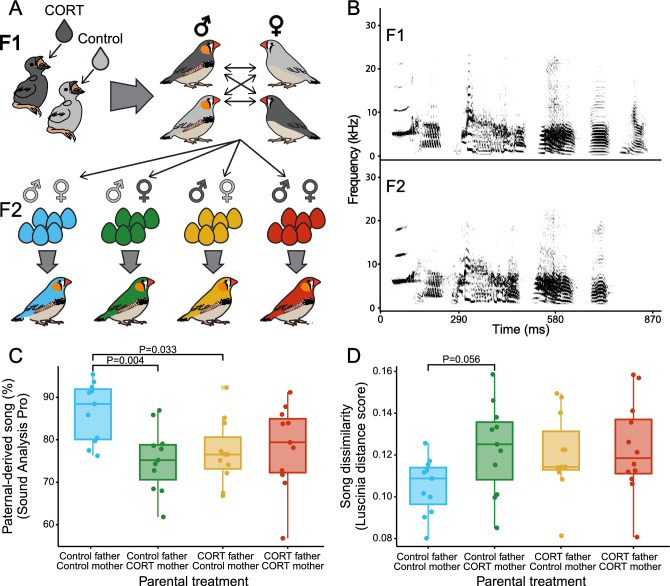
(**A**) One generation of wild-derived zebra finch nestlings (F1) was administered a CORT treatment (dark gray) or control vehicle (light gray). When adult, birds bred in an aviary with a balanced distribution of control and CORT-treated males and females, with the resulting generation having either one, two, or no CORT-treated parents. Once their male offspring (F2) reached adulthood we performed an experiment to measure song copy accuracy and IEG expression in the brain following tutor or novel song playback. (**B**) Example spectrograms of song motifs from one father (F1 male) and his son (F2 male). (**C**, **D**) Parental treatment and the similarity between the F2 male’s songs to their social father’s song as determined by (**C**) the % paternal-derived song (Sound Analysis Pro) amd (**D**) song distance score (Luscinia). P-values for panels (**C**) and (**D**) were generated using emmeans post hoc tests.

We found that parental treatment influenced how much of the F2 male’s song was copied from their social father (i.e., % paternal-derived song, paternal treatment*maternal treatment, *X*^2^ = 12.001, P = 0.007). Post hoc tests revealed that males with two control parents copied tutor songs more closely, with a higher % paternal-derived song, compared to males with a CORT-treated mother and a control father (emmeans, P = 0.004, Fig. 1C) or a CORT-treated father (emmeans, P = 0.033, Fig. 1C). However, we found no effect of parental treatment on the % copy completeness (paternal treatment*maternal treatment, *X*^2^ = 0.486, P = 0.902). When testing the song dissimilarity between F2 males and their social father (i.e., Luscinia distance score), we did not find evidence of an interaction effect of parental treatments on song dissimilarity (paternal treatment*maternal treatment, *X*^2^ = 4.798, P = 0.187), but we found that males with CORT-treated mothers and control fathers sang songs that were less similar to the songs of their social father (emmeans, P = 0.056, Fig. 1D). In other words, the offspring of CORT-treated females sang songs that consisted of a higher percentage of non-paternal sounds and were less similar to their social father’s song overall.

To further examine the tutor-tutee relationship between social fathers and their offspring, we also tested how closely the songs of the F2 males resembled the songs of their social fathers compared to the other F1 males in the breeding colony (i.e., all other potential tutors). We converted the song distance score to Euclidean coordinates^43,44^, but we did not observe a close association between the songs of the F2 males and their social father in Euclidean space (supplementary materials), indicating that the F2 males did not sing songs that were more similar to the song of their social father than to other potential tutors. To test whether F2 males were more or less likely to copy their song from males from different treatment groups, we then ranked all potential F1 tutors of each F2 male according to the song distance score, and we tested whether the “tutor rank” of the social father differed between treatments. While we found that there appeared to be some differences between the groups, the differences were not statistically significant (supplementary materials).

### IEG expression and playback similarity

Contrary to our prediction, there were no strong directional effects of playback type on IEG expression dependent on parental treatment in either brain region (supplementary materials). This indicates that there were no simple F1 treatment-mediated directional impacts on F2 IEG responses to putative tutor playback. As the IEG response is a function of the saliency of the stimulus, one reason for this might be that the treatments affected vocal learning dynamics within the colony, thereby altering the saliency of the tutor song. Consequently, we sought to test whether IEG expression levels in response to playback were affected by treatment depending on the degree of similarity between the F2 male and the playback song. To do this, we examined gene expression levels across the song distance score between the F2 male’s song and the playback song and defined this as the “playback song dissimilarity”. This approach tests whether the parental treatment affects the IEG response to playback while considering how similar the playback song is to what the F2 males produced.

**Figure 2:**
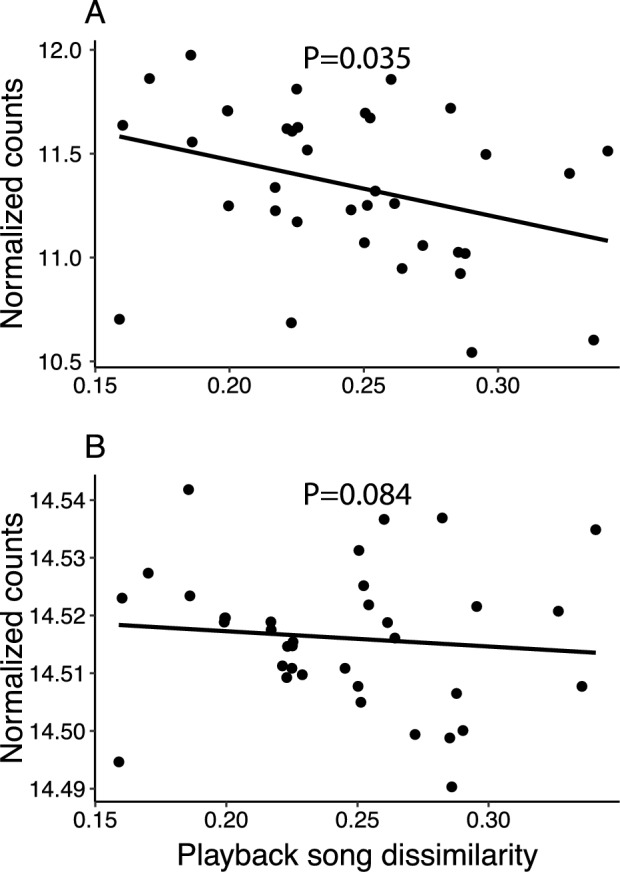
(**A**) Expression of ARC, and (**B**) NR4A3 in NCM in relation to the dissimilarity between the playback song and the male’s own song (playback song dissimilarity). Points represent normalized data for each individual who was extracted from an intercept-only MCMC.qpcr model. Both ARC and NR4A3 expression decreased with playback song dissimilarity (i.e., dissimilarity to the male’s own song). P values are model results for the main effect of playback song dissimilarity.

**Figure 3:**
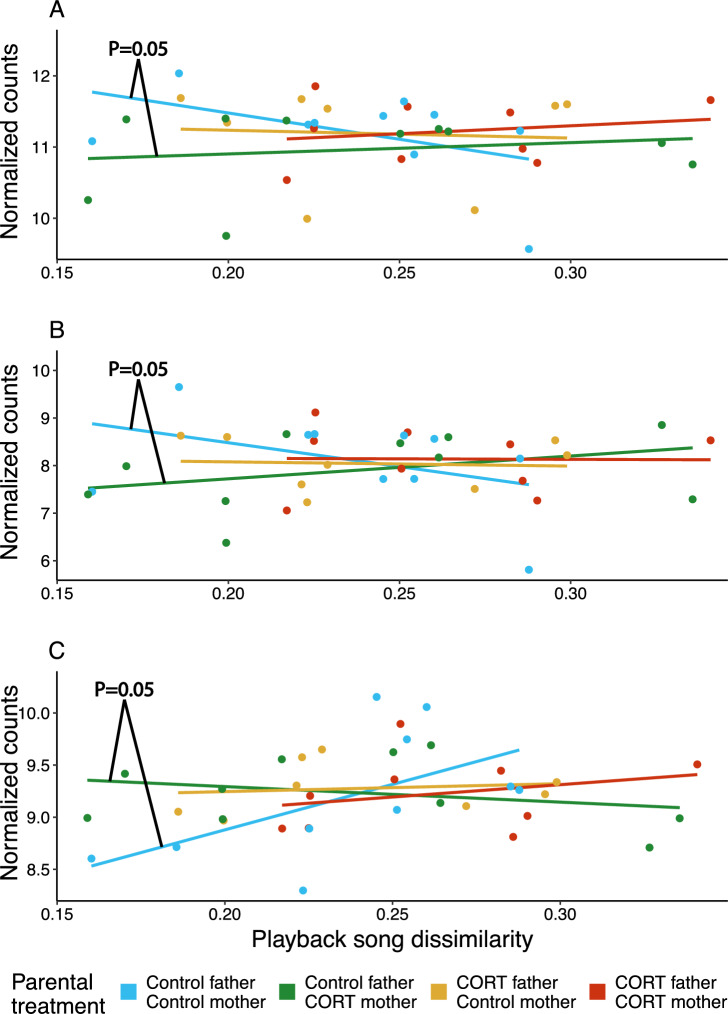
Expression of (**A**) ARC, (**B**) CFOS, and (**C**) BDNF in CMM, in relation to the dissimilarity between the playback song and the male’s own song (playback song dissimilarity), plotted for each parental treatment group. Points represent normalized data for each individual who was extracted from an intercept-only MCMC.qpcr model. For all three genes, birds with a CORT-treated mother showed the opposing expression pattern to birds with two control parents (P values represent this difference). Absolute CFOS expression showed a slight decrease with playback song dissimilarity, while BDNF increased with playback song dissimilarity.

In NCM, ARC expression in response to playback was negatively associated with increasing dissimilarity between the playback song and the male’s own song (MCMC.qpcr, playback song dissimilarity, Mean = − 25.234, P_adj_ = 0.035, Fig. 2A). This continuous relationship is consistent with a previous finding that ARC expression is higher in response to a two-group comparison of tutor song and novel song^39^. We also observed a similar non-significant effect of decreasing NR4A3 expression (MCMC.qpcr, playback song dissimilarity, Mean = − 19.404, P_adj_ = 0.084, Fig. 2B), with a higher playback song dissimilarity. In CMM, both ARC and CFOS tended to decrease with increasing playback song dissimilarity in males from two control parents, but the effect was not statistically significant (ARC: Mean = − 14.856, P_adj_ = 0.143; CFOS: Mean = − 20.120, P_adj_ = 0.060, Fig. 3). In contrast, BDNF showed a marginally significant increase in expression with increasing playback song dissimilarity in males with two control parents (MCMC.qpcr, playback song dissimilarity, Mean = − 21.246, P_adj_ = 0.050, Fig. 3), which is consistent with the results when simply comparing tutor vs novel song playback (Table S4, supplementary materials). In other words, we found weak support that these genes were differentially expressed depending on the similarity between the male’s song and the song to which they were exposed, regardless of whether the playback consisted of tutor or novel song.

When comparing treatment groups, we found weak evidence that the expression of ARC, CFOS, and BDNF depended on an interaction between parental treatment and playback song dissimilarity (Fig. 3, Supp Table S5). Males with two control parents showed a contrasting pattern from the other treatment groups, but the difference was only marginally significant when compared with males with a CORT-treated mother and a control father (CFOS: Mean = 27.295, P_adj_ = 0.05; ARC: Mean = 22.547, P_adj_ = 0.05; BDNF: Mean = 24.268, P_adj_ = 0.05). For BDNF expression, the pattern observed closely resembled the results observed in the models with two playback types, where novel song elicited stronger BDNF expression in males with two control parents only. See supplementary Table S3 for the full model output.

### Extra-pair parentage (EPP)

We obtained genotypic data to compare 42 pairs of F2 males and their social fathers, and 39 pairs of F2 males and their social mothers. From these pairs, three males were confirmed as EPP on the father’s side, indicating an extra-pair paternity percentage of 8%. There was no confirmed extra-pair parentage on the mother’s side. If we included unassigned (not confirmed) males as EPPs, the rate for extra-pair paternity was 14% and the extra-pair maternity was 5%. See supplementary table S6 for more details.

## Discussion

Developmental conditions have been posited to alter trait expression across generations through a combination of cultural and genetic mechanisms^3,4^. Here, we sought to test the intergenerational impact of early-life stress on vocal learning. In songbirds, vocal learning is determined by the social environment, with both genetic and environmental influences, and song production has large-scale consequences for both individual fitness and population-level outcomes^45,46^. We show, for the first time, that stress during parental development can cascade to affect song learning in the next generation and the consequent gene expression response to song playback. Specifically, we found that males with either a CORT-treated mother or father copied their putative tutor less well, but this was not true when both parents received the CORT treatment. These effects on IEG expression and song acquisition may have been mediated through differences in social learning strategies when living in large colonies, with many potential tutors^47^.

A fundamental theory for the understanding of how song can function as an honest signal of male quality is that the songs males have learned reflect the conditions they experienced during early development^48,49^. Adverse developmental conditions can reduce male quality by decreasing growth and development^26,50^ and can cause compensatory growth with negative fitness consequences^51^ (but see^52^ for a discussion of positive fitness consequences). For example, neural growth in early development is vital for behavioural development and effective cognitive function^15,16,53^, and compensatory neural growth can affect longer-term cognitive performance^54^. Consequently, within a generation, developmental conditions have previously been shown to directly affect neural development^15,55^, song learning^26,56^, and IEG expression following tutor song playback^39^. There are, however, also examples of studies that have found no or a positive relationship between measures of developmental stress and song learning and other cognitive performances (e.g.^21,57^). In zebra finches, developmental conditions can also affect song learning and social associations during foraging^58,59^, suggesting that neural growth alone cannot explain the effects of developmental conditions on song learning. One study confirmed the association between locomotor activity during the sensitive period of song learning and vocal development, as individual males that moved more demonstrated better song copying and longer songs^60^. Although we know that developmental conditions can be powerful drivers of intergenerational effects^61,62^, these studies suggest that the intergenerational impacts of developmental history on song learning are likely complex and possibly due to multiple interacting mechanisms.

We found that developmental treatment had intergenerational effects on song learning. The most parsimonious explanation for this finding is that within a captive colony of breeding zebra finches, parental experience affects offspring song learning, and consequently the response to parental song. IEG expression in the NCM and CMM following song playback is related to the salience of the stimulus, but it is also related to the novelty of the stimulus^31,32,34,35^. There are therefore several non-mutually exclusive explanations for how CORT treatment may affect offspring song memory. For example, changes in tutoring interactions by the F1 males, tutor preference of the F2 males, or the ability of F2 males to incorporate elements from several tutors into their song could all potentially affect the neurogenetic response to song playback. The exact mechanism for this effect is unclear, but our data are consistent with the suggestion that adverse parental developmental experiences reduce the likelihood that a social father acts as an effective tutor when breeding in a colony environment. However, we did not find a difference in either song similarity measure between male offspring of two control parents and those of two CORT treatment parents, suggesting either the power to detect effects on song learning is low, or the father-son tutor interactions also depend on the mother. We deliberately used a breeding colony in our experimental design, as this is similar to how zebra finches breed in the wild^47^, meaning that the conditions the birds in this study experienced mimic the conditions wild zebra finches would experience. Studies that have examined song learning in colony-living zebra finches have found that young males tend to learn the songs of their social fathers (or males with songs similar to their father) but also aggressive males and the males they associate with most^63–66^. Indeed, in zebra finches, song production has a low heritability level and is mainly controlled by environmental factors^67,68^. Together, these findings suggest that the developmental conditions of the parents affect both the dynamics of vocal learning and song production of their offspring. One recent, relevant study examining the impact of prenatal acoustic stimuli using the same colony of zebra finches showed that prenatal exposure to a sound treatment altered the incorporation of nonparental syllables into the song, suggesting a possible change in tutoring interactions^69^. It is therefore likely that our results in terms of both song acquisition and brain gene expression are, at least in part, indicative of changes in social tutoring interactions.

Linked to the changes in tutoring interactions, our results indicate that parental (and particularly maternal; F1) developmental conditions affect IEG expression following song playback in adult F2 offspring. Our previous research has shown that tutor song playback elicits higher ARC expression than novel playback, and this response was affected by a developmental stress treatment^39^. Here, we observed that NCM ARC expression was higher in response to songs that were similar to the male’s own song, which reflects these previous findings in part. Additionally, the negative association between CFOS expression and playback song dissimilarity is in line with previous research by Bolhuis et al.^33^, who showed a positive association between the strength of CFOS expression following tutor song playback and how closely the male’s song resembled that of the tutor. Hence, some of the patterns observed here follow what has previously been shown, but no study has previously found any evidence that IEG expression following song exposure can be affected by parental developmental conditions.

We found that males with a CORT-treated mother and a control father showed contrasting immediate early gene expression patterns compared to males with two control parents but that crucially, this relationship depends on the salience of the stimulus. Interestingly, for males with either one or two CORT-treated parents, there was no apparent IEG expression change (or a slight increase in ARC or CFOS expression) with playback song dissimilarity, suggesting that parental treatment influences not only song learning but also the response to paternal song. The difference in gene expression change with playback song dissimilarity between treatment groups was only statistically significant when comparing males with a CORT-treated mother and a control father and males with two control parents, mirroring the results observed for song similarity between F2 males and their social fathers. As with the effects on song copy accuracy, the drivers for the effect of parental treatment on IEG expression are unclear. One possible (but speculative) explanation is that F1 developmental treatment directly affected tutoring effort, behaviour or family dynamics, such that F2 tutees learn from other males in the colony.

Maternal treatment had a stronger effect than paternal treatment on both offspring song production and IEG expression following song playback (although there were statistical interactions between paternal treatments for some variables). However, this was not true when a CORT-treated female was paired with a CORT-treated male. When considering a range of variables, males with two control parents were most often distinguishable from the males with a control father and CORT-treated mother. Additionally, song dissimilarity between the F2 males and their social father was affected by maternal but not paternal treatment or the combination of parental treatments, implying that maternal effects may be stronger than paternal effects in this system. Song copy accuracy is a condition-dependent trait in zebra finches^70^, but this is the first study to indicate that the early developmental condition (which was affected by the cort treatment, see *50*) of the parent may also affect this learning process. Zebra finch females tend to mate assortatively based on condition or developmental background, and lower-quality females have been shown to prefer songs of lower quality^71–73^. It is possible that the F1 CORT-treated females were more likely to pair with males who sang lower-quality songs, which could affect the tutoring relationship between the F2 males and their social father. Female birds can also directly affect the physical development of their offspring through hormones and nutrients deposited in the egg^74,75^. Females with elevated CORT levels during ovulation can deposit more CORT into the egg, which can have lasting effects on the physiology of the offspring^76,77^. The CORT treatment had lasting effects on the morphology of the F1 birds^50^, and a similar treatment is known to have lasting (but not lifelong) effects on blood CORT levels^78^. When we examined circulating F2 CORT levels in a separate study, we found effects of paternal, but not maternal, treatment^79^. However, we also found that maternal body mass was correlated with offspring body mass and that this correlation was affected by maternal treatment^79^. It is therefore possible that the effects on song learning were mediated through maternal effects on offspring growth and body condition. Physiological differences between CORT-treated and control females does not, however, explain why effects were not observed in F2 birds with two CORT-treated parents.

Our results suggest that several of the traits we examined may have been influenced by both maternal and paternal developmental conditions. The zebra finch is an altricial species with biparental care^80^. In many species, deprivation of parental care can be a driver of intergenerational effects^81,82^. It is also possible that the effects of maternal and paternal treatment are mediated by epigenetic inheritance^8,83^. We would need to know the biological parents of the F2 males to draw any conclusions as to whether epigenetic mechanisms may mediate the effects observed in this study. Our results from a subset of F2 birds indicate that there is a small number of extra-pair offspring in the cohort of birds used in this study. While we used a paired design with male siblings to control for both genetic effects and the song structure and song quality of the social father, we cannot rule out that genetic parentage could have had an effect on the results. Future experiments should use cross-fostering or paternity analyses to test whether genetic, prenatal, or postnatal effects are the drivers of intergenerational effects on song learning and gene expression.

Our study shows that a CORT treatment experienced in one generation can affect song learning, plausibly through altered tutoring interactions and consequently the gene-expression response to song playback in the next generation, although further studies are needed to determine the exact underlying mechanisms. At the population level, alterations in song copying as a result of parental developmental history could impact population divergence and dialect formation. Biases for tutees to copy from several potential tutors may provide a mechanism for individuals to prevent being detrimentally impacted by their parents’ developmental histories, providing population stability^5^. We have shown that environmental conditions have longer-term effects on vocal learning than previously thought. This may have implications for the evolution of song as a condition-dependent social signal, as song production reflects not only the individual’s own developmental history but also that of the previous generation.

## Methods

### F1 experimental treatment and breeding experiment

We dosed F1 nestling zebra finches with either CORT or control treatments during early development (see Supplementary materials). This treatment reduced F1 body mass and body size during the nestling stage and into adulthood, indicating that the treatment negatively affected body development^50^. At sexual maturity (post-hatch day, PHD > 100), they bred in two separate aviaries (N_Control_ = 9 females, 10 males; N_CORT_ = 9 females, 10 males per aviary). At approximately PHD 70 (+/−5 days), after the sensitive period for song template acquisition, we moved the F2 offspring into a separate room with single-sex cages (100 × 50 × 50 cm) until song playback testing. We used social parentage to determine the treatment group of the F2 birds, as we were not able to perform full paternity analyses in this study. For methodological details, see the supplementary materials and Kraft et al.^50^.

### Song analysis

We recorded all F1 males before breeding and all F2 males after they reached sexual maturity (PHD > 100). All song analyses were performed by a single observer (FLK), and the observer was blind to the treatment and relatedness of the males. Although zebra finch males produce a single stereotypic song (i.e., one song type per male), we used multiple song instances (motifs) per male for the analyses to account for individual variation in song performance.

Using Sound Analysis Pro 2011^84^, we compared five song motifs from each F2 male and their social father using the similarity batch function in SAP2011 (25 comparisons per father-son pair: N = 1100 comparisons). The similarity score is based on how many sounds (the specific terminology used by the software designer, not specifically linked to motifs or syllables) are copied from a reference song motif to another song motif. We used both the social father’s (the usual procedure) and the F2 male’s (a novel approach) song motifs as the reference song motif, and we refer to these similarity measurements as “% copy completeness” and “% paternal-derived song”, respectively (we consulted with the creator of the software before performing this analysis). Paternal-derived sounds are copied from the father, and nonpaternal sounds could be a result of innovation, learning from other tutors in the colony, or unsuccessful copies^64^. The % copy completeness represents how much of the social father’s song has been successfully copied into the F2 male’s song. The % paternal-derived song represents how much of the F2 male’s song has been obtained from the social father’s song. It would therefore be possible for a father-son pair to show high % completeness and low % paternal-derived song if most of the father’s song, as well as song elements from other tutors, were incorporated into the F2 male’s song.

To better understand whether any differences in song similarity between treatment groups were a result of the F2 males differing in their social learning strategy (i.e., tutor choice), we performed further song similarity analysis using Luscinia (http://rflachlan.github.io/Luscinia/). Lusinia performed better for comparisons between large numbers of songs and individuals compared to the SAP2011 batch function. We compared the songs of each F2 male to all potential F1 tutors in their home aviary using five song motifs per individual and the dynamic time-warping method^44^. We did not consider horizontal transmission of song, as we did not have reliable information about which F2 males were housed together after PHD 70. The total number of F1 male to F2 male song comparisons was 19,825 (aviary one: 7225 comparisons of five songs each from 17 F2 males and 17 F1 males; aviary two: 12,600 comparisons of five songs each from 28 F2 males and 18 F1 males). We used the same parameters as previously described in Boogert et al.^58^, but we compared whole song motifs using the knitted method instead of individual element comparison. We generated a similarity matrix for all songs and used the song distance score from this matrix to test the song dissimilarity between pairs of song motifs (a higher song distance score indicates that the songs are less similar). We used the song distance score between the songs of each F2 male and the songs of their social father to test whether parental treatment affected this measure of song similarity (n = 1125 comparisons across five song motifs from 45 F2 males and five song motifs from their social father). We refer to the distance score between F2 males and their social father as “song dissimilarity”.

### Playback experiment, RNA extraction, and quantitative PCR

We tested the response of 45 F2 males to song playback at PHD 101–108. Sets of full social siblings were exposed to 40 to 50 min of a novel song or their social father’s song in a paired design. The novel song consisted of recordings from a randomly selected male from the opposite breeding aviary to the target F2 male. See supplementary materials for more details on how the playback files were created. After the playback had finished, the male was immediately euthanized, the brain was removed, and the two hemispheres were stored at − 80 °C before being shipped to Queen Mary University of London, UK, for sectioning.

We used a cryostat to cut 12 100 µm thick sagittal sections per hemisphere per male, and from these sections, we collected tissue punches in both the NCM and CMM. We extracted RNA from the tissue punches and quantified gene expression using quantitative real-time PCR (qPCR) on the target genes ARC, BDNF, EGR1 (ZENK), CFOS, and NR4A3 and the housekeeping genes HPRT, PGK1, and YWHAZ. The target genes were selected based on previous studies that have linked these IEGs to increased neural activity and memory formation in songbirds^30,42^. For details on this method and the primers and probes used, see the supplementary materials, Fig. S1, and Table S1.

### Estimation of extra-pair parentage

For the offspring, genomic DNA was obtained at the time of RNA extraction. For potential parents, genomic DNA was isolated from clotted whole blood samples stored in 70% ethanol. Primers were designed to amplify 10 genomic fragments previously selected for microsatellite analysis^85^, and these amplicons were instead used to prepare indexed libraries for sequencing via Illumina MiSeq^86^. BWA v0.7.17-r1188 was used to align quality trimmed FASTQ sequences to the zebra finch reference genome (bTaeGut1.4.pri), and allelic variants (including SNPs and insertions/deletions) were identified and individuals genotypes were assigned using GATK-Haplotype Caller v4.5.0.0. Parentage was modelled using COLONY software v2.0.7.1. For details on this method and the gene-specific and index primers used, see the supplementary methods and Tables S2, S3.

### Statistical methods

All statistical analyses were completed using R version 4.2. We tested for effects of parental CORT treatment on the similarity between the songs of the F2 males and their social fathers (Sound Analysis Pro: % copy completeness and % paternal-derived song; Luscinia: song dissimilarity) using linear mixed models and the lme4 and emmeans packages^87,88^. The models included aviary ID, maternal treatment, paternal treatment, and the interaction between maternal and paternal treatment as fixed factors. Father ID was included as a random factor to account for relatedness effects, and individual ID was included to account for repeated measures. We estimated the statistical significance of interaction effects using likelihood-ratio tests of the full model compared to a model without parental treatment. We used emmeans pairwise post hoc comparisons to test for treatment differences in song copying accuracy.

We tested for the effects of parental CORT treatment on offspring IEG expression using the MCMC.qpcr package^89^. We created informed models with soft normalization (30,000 iterations, thinning interval 10, burn in 7000). The housekeeping genes HPRT, PGK1, and YWHAZ were used as control genes. We created one model per brain region, and we included father ID as a random effect to account for relatedness and the paired playback experimental design with siblings. The fixed effects were parental treatment, a four-level factor with the levels “control father and control mother”, “control father and CORT-treated mother”, “CORT-treated father and control mother”, and “CORT-treated father and CORT-treated mother”. The models also included playback type (tutor and novel) and the interactions between these factors. Control treatment (control father and control mother) and novel playback were set as reference levels, and we adjusted for multiple comparisons using the false discovery rate method.

As there were no clear effects of playback type on IEG expression, we also created two MCMC.qpcr models with the playback song dissimilarity between the song of each F2 male and the playback song to which they were exposed. We were thus able to test for changes in IEG expression depending on a continuous measure of song similarity. We used a subset of samples (N_NCM_ = 36 and N_CMM_ = 37) for which we had both song recordings and IEG expression data. Apart from the replacement of playback type with playback song dissimilarity, the models were identical to those above.

### Supplementary Information

Supplementary Information.

## Data Availability

The datasets used and analysed in this study are available from the corresponding author on reasonable request.
