# Supplementary material for: Parental developmental experience affects vocal learning in offspring
Source: Sci Rep. 2024 Jun 14;14:13787. doi: 10.1038/s41598-024-64520-8 (PMC11178867; doi:10.1038/s41598-024-64520-8)
Supplement: Supplementary file 1 — Supplementary Information. [file 41598_2024_64520_MOESM1_ESM.docx]

# Supplementary materials

## Supplementary methods

## **F1 experimental treatment**

In 2015 at Deakin University, Australia, we dosed nestling zebra finches (F1) with corticosterone (the main avian glucocorticoid). From five to 18 days post-hatching, CORT-dosed nestlings received 0.25mg/ml of CORT solution (Sigma Aldrich; 25 µl CORT dissolved in peanut oil) orally, twice daily. Control nestlings received 25 µl of peanut oil vehicle only, twice daily. This dose level elevates CORT levels in nestlings to biologically relevant CORT levels, as documented in wild zebra finches (Crino et al., 2018; Spencer & Verhulst, 2007). This treatment reduced F1 body mass and body size during the nestling stage and into adulthood, indicating that the treatment negatively affected body development (Kraft et al., 2019). In a separate study, we also found that F2 birds with CORT-treated fathers had elevated circulating CORT levels, and the correlation between maternal body mass and offspring body mass and affected by maternal CORT treatment (Kraft et al., 2021).

**Breeding experiment**

Once the F1 birds reached sexual maturity (post-hatch day, PHD>100), we conducted a breeding experiment with 72 birds in two separate aviaries (3x2x2m; each aviary contained N_Control_=9 females, 9 males; N_CORT_=9 females, 9 males). Nesting material (shredded burlap and dry grass) and 24 nestboxes were provided in each aviary. During breeding, birds had *ad libitum* access to a commercial seed diet, water, cucumber, grit, spinach, and egg. We housed birds on a 14:10 light/dark cycle at 20ºC (+/- 1 ºC) with 50% humidity. Birds could pair and breed freely over sequential breeding attempts, and nests were inspected daily. Halfway through the breeding experiment (April 2018), we swapped the females in the two aviaries, so that the two aviaries would form one population. We removed the nest boxes before doing so, and we did not abort any active clutches. Around PHD 70 (+/- 5 days), we moved the F2 birds to single-sex holding cages (100x50x50 cm) and they were maintained on the same light cycle and humidity as above until song playback testing.

## **Recording F2 males**

We recorded all F1 males before breeding, and we recorded all F2 males after they reached sexual maturity (PHD>100). We moved males to a separate recording room where they were given 2-3 minutes to acclimatise. The recording session began when we introduced a companion female into the cage and lasted for five minutes if the male sang, or up to 10 minutes if the male did not sing. If a male did not sing for the first five sessions, we attempted to record it over a total of 10 sessions before excluding it from the analysis. We recorded using an H6 handy recorder (Zoom Corporation, Japan), and an MKE 2P condenser tie-clip microphone (Sennheiser Electronic GmbH, Germany).

## **Song analysis**

We analysed the F1 and F2 songs using Sound Analysis Pro 2011. Using Adobe Audition CC 2018, we cut five songs per individual from at least two different recordings, and we applied a high pass filter at 500Hz to remove background noise (sampling rate at 44 kHz).

The song motifs were selected by observing an entire song bout and picking the most common version of the song motif that had little or no background noise and no female calls in the background. Neither the first nor the last song motif of a bout was selected, to avoid introductory notes affecting the analyses. We selected song motifs from different song bouts when the male sang several song bouts of adequate clarity and length. As there were not always clear silent gaps in the songs, we marked syllables as distinct when there was a clear shift in frequency and shape, or if syllables were separated by gaps of silence.

Using SAP2011, we estimated the duration, number of syllables, and pitch (fundamental frequency) of five songs per individual (N=220 songs from 44 F2 males from 16 fathers). The song parameters were estimated automatically, and we counted the number of syllables manually by observing sonograms. All songs were analysed by the same researcher to avoid observer bias.

We also analysed song similarity using Luscinia and dynamic time warping. The acoustic features included in this analysis were based on Boogert et al (2018) and included: time, mean frequency, fundamental frequency, normalised fundamental frequency, fundamental frequency change, and Wiener entropy. All features were weighted equally except for time, which was weighted as 10. We used compression factor: 0.001 and minimum element length 10. The maximum dynamic time warp of 100% was allowed, frequency parameters were log-transformed, and elements were weighted by their relative amplitude. We selected dynamic warping and interpolation in time warping. We used five time-alignment points.

## **Song similarity within breeding experiment rooms**

The F2 males used in this experiment were produced through a breeding experiment in two breeding aviaries, and they were moved into a separate room with single-sex cages after the sensitive period for song template acquisition. The males could therefore acquire the song template from several F1 males. To better understand whether F2 males tended to sing songs that were more similar to the songs of their social father compared to other nearby males, we plotted the song similarity of all F1 and F2 males in Euclidean dimensions. We used the Luscinia function “non-metric multidimensional scaling” to convert the dissimilarity matrix used for song similarity analyses into Euclidean coordinates in two dimensions. We used the same dataset and acoustic features for this analysis as the ones described above.

**Father tutor rank**

As we did not see a strong association between the songs of the social fathers and their male offspring, we wanted to test how the social fathers ranked as song tutors. We ranked all potential tutors for each F2 male based on the distance score between each pair of individuals, with the top-ranked F1 male being the one with the most similar song to each F2 male. We then extracted the rank that the social father received for each of the F2 males to create a data set of father tutor rank (n=45 F2 males). Father tutor rank was therefore lower for F2 males who sang songs that were more different to their social fathers and more similar to other potential tutors.

As very few social fathers had the top tutor rank (i.e. the most similar), we instead focused on whether the social father was in the top half of all potential tutors. We split the social father into two groups based on whether they fell within the top or bottom 50% of song similarity to each F2 male (“Top-ranked” and “Bottom-ranked”). Top-ranked social fathers were amongst the top 50% of F1 males in terms of song similarity with their F2 offspring (within the room the F2 male was born in). We then performed a chi2, where the chi2 tables contained the treatment groups (parental CORT treatment) and the number of top-ranked and bottom-ranked social fathers.

## **Playback experiment and tissue collection**

We tested the response of 45 F2 males to song playback at PHD 101-108. Sets of full siblings were exposed to either novel or their father’s song in a paired design. The novel song consisted of recordings from a randomly selected male from the opposite breeding aviary to the target F2 male. Each playback file (novel and social father) consisted of repeats of two to three seconds of song (from at least five different song recordings) followed by 10 seconds of silence. To avoid differences in background noise and playback loudness confounding the effect of the song playback, we applied a high pass filter (500Hz) and matched the loudness to a total RMS of -25dB. We adjusted the loudness of the playback to 75-85 dB using a sound level meter held ca 50 cm from the speaker. We moved individual males to a separate room the day before testing, and we initiated song playback remotely. After 40 to 50 minutes of playback, the male was immediately euthanised, the brain extracted, and the two hemispheres were separated along the midline and flash-frozen. The 40-50 minutes of exposure was chosen to get close to the peak exposure time for EGR1 (Mello & Clayton, 1994; Mello et al., 1992). The hemispheres were stored at -80 ºC and shipped to Queen Mary University of London, UK for sectioning.

## **RNA extraction and quantitative PCR**

We sectioned each hemisphere using a Leica Model CM3050S cryostat (Leica Biosystems). We collected 12 100µm sagittal sections per hemisphere starting at the midline of the brain. We collected one 2mm brain tissue punch (Stoelting, catalogue no. 57405) in both the NCM and CMM on each sagittal section (supplementary materials Fig. S1) by punching the area superior and anterior to field L (identified as a thin pink line). We extracted RNA from the tissue punches using Qiagen AllPrep® DNA/RNA Micro Kit (catalogue no. 80284) in accordance with the kit instructions. We used a 26G syringe to draw the sample up and down to disrupt the tissue before using the vortex recommended in the protocol. For cDNA synthesis, we used the Qiagen QuantiTect Reverse Transcription kit (catalogue no. 205311) in accordance with the kit instructions.

We quantified gene expression using quantitative real-time PCR (qPCR) on cDNA from both NCM and CMM, the target genes ARC, BDNF, EGR1 (ZENK), CFOS, and NR4A3, and the housekeeping genes HPRT, PGK1, YWHAZ. The target genes were selected based on previous studies that have linked these IEGs to increased neural activity and memory formation in songbirds (Clayton et al., 2019; Warren et al., 2010). A list of the primers and probes used is given in supplementary materials (Table S1). All qPCR reactions were run in triplicate along with no-template controls, on a CFX Connect real-time PCR detection system (Bio-Rad). All reactions were prepared with 15ng template cDNA per well and the appropriate supermix (Biorad iTaq Universal SYBR Green Supermix for primer-only assays, 5x HOT FIREPol Probe Universal qPCR Mix for assays with hydrolysis probes). For all assays, the optimal annealing temperatures were between 60-61^o^C. When there was a clear outlier in one of the three runs, or the melting profile (for SYBR Green assays) of a sample indicated off-target amplification, we excluded the sample from the analysis. The final sample sizes were N_NCM_=44 and N_CMM_=45.

## **Estimation of extra-pair parentage**

Offspring DNA was isolated from brain tissue punches at the same time as RNA was extracted using the Qiagen AllPrep® DNA/RNA Micro Kit (catalogue no. 80284). For potential parents, genomic DNA was isolated from clotted whole blood samples stored under 70% ethanol, following a published protocol (Qamar et al., 2017). The supernatant was removed and replaced with 500uL lysis buffer (1% SDS, 100mM Tris-Cl, 200mM NaCl, and 2mM EDTA), 490uL filtered distilled water and 10uL of 20mg/mL proteinase K. The mixture was homogenized by gentle vortexing and incubated for 2 hours at 50°C in a heat block, followed by shearing with a 20 gauge blunt end needle affixed to a 1mL syringe. DNA was recovered via precipitation with sodium acetate and ethanol, followed by resuspension in Tris EDTA buffer (pH 7.5), with incubation at 37C for 30 minutes to resolubilize the DNA. DNA concentration was measured with Nanodrop spectrophotometer (Thermo Fisher Scientific)

Amplification of DNA fragments and library prep was adapted from a published method (Nag et al., 2017). 10 primer sets previously optimized for microsatellite analysis (Dawson et al., 2010) were modified to append upstream header sequences that are incorporated during the first PCR reaction (gene specific primers, Table S2). Index primers (Table S3) included sequences that anneal to the gene specific products with added overhangs incorporating both unique barcodes and annealing sites for Illumina sequencing primers. PCR primers were purchased from Eton BioScience. All PCR reactions were performed with Multiplex 5X PCR Mastermix (New England Biolabs).

Genomic DNA from each individual (offspring or parent) was amplified first with gene specific primers, in multiplex reactions (Multiplex 1: Tg02-078, Tg12-015, Tg13-017; Multiplex 2: Tg01-124, Tg05-046, Tg07-022; Multiplex 3: Tg03-098, Tg04-012, Tg08-024, Tg22-001). Expected product sizes were confirmed with TapeStation automated electrophoresis (D1000 ScreenTape assay, Agilent, California USA). Products for each individual were pooled, purified with AMPure XP beads (Beckman Coulter, California, USA), then amplified in a second PCR reaction with a unique combination of index primers. The resulting amplicons were normalized and pooled, and submitted to the Clemson University Genomics and Bioinformatics Facility for library prep and sequencing (Illumina MiSeq v3, 2x300bp, 600 cycles). Data were returned as demultiplexed FASTQ files for each of 50 offspring and 65 potential parents. These raw sequences were quality-trimmed with TrimGalore (--paired -j 7 --quality 28 --phred33 --nextera --fastqc --gzip --length 150), and aligned to the zebra finch genome (bTaeGut1.4.pri) with bwa-mem (v0.7.17), using default settings in paired-end mode. BAM alignment files were processed with GATK-Haplotype Caller to identify genotypic variants. Variant calls were filtered for a quality score of 1000 or more, resulting in 88 markers with at least partial coverage among the offspring and potential parents. These genotypes were used to model parentage in COLONY software v 2.0.7.1 , using social parents as priors for presumed parentage and for estimating sibship size, allowing polygamy for both fathers and mothers, and excluding parentage for more than two mismatches between offspring and parent.


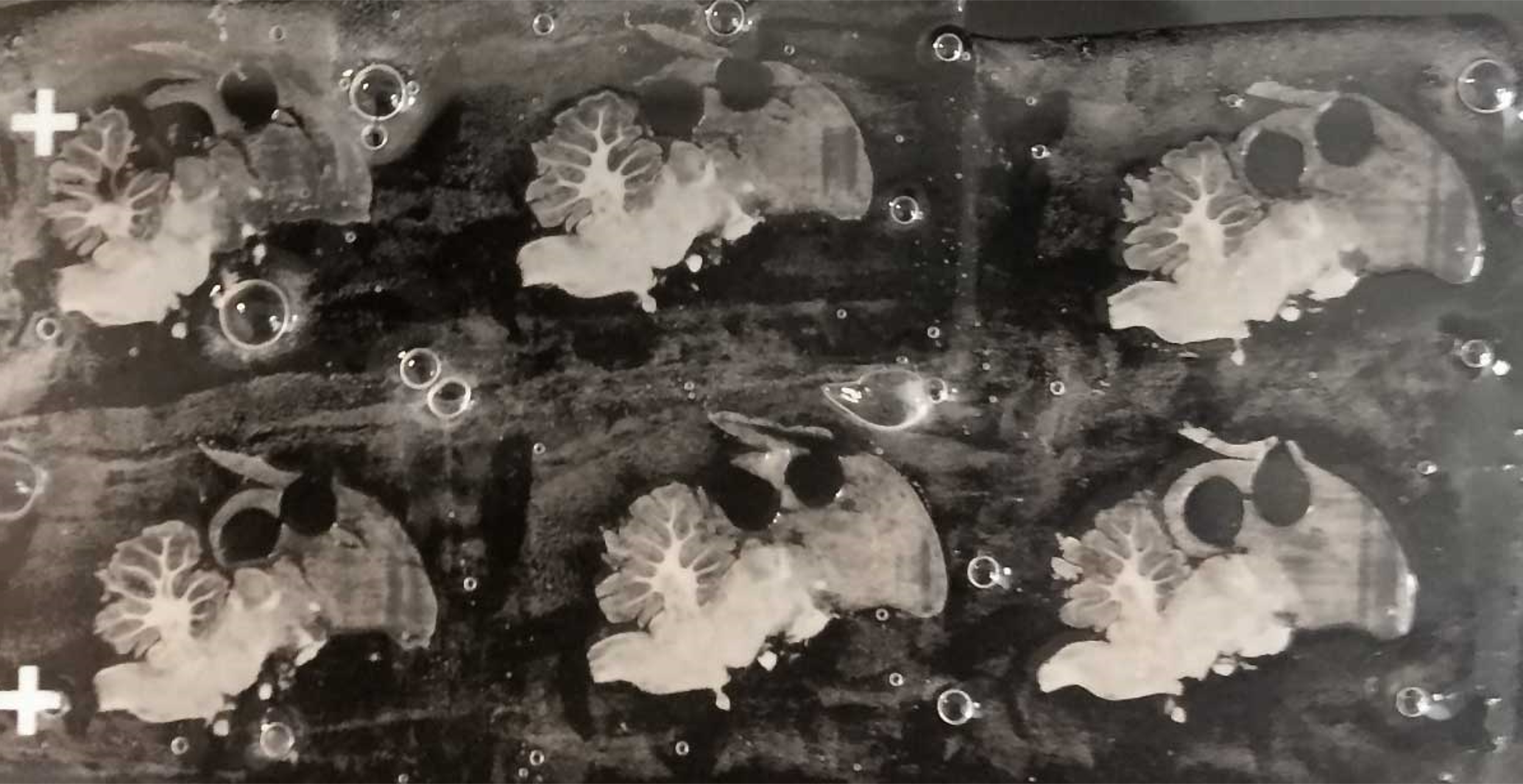


Fig S1. Cryosections of the zebra finch brain (100 µm) on a glass microscopy slide, with two tissue punches taken out of the NCM and CMM regions.

*Primers and probe sequences*

Table S1. Primers and probe sequences for qPCR measurements of gene expression.

| **Target gene** | **Forward primer** | **Reverse primer** | **Hydrolysis probe** |
| --- | --- | --- | --- |
| YWHAZ | GTGGAGCAATCACAACAGGC | GCGTGCGTCTTTGTATGACTC | SYBR-green assay |
| CFOS | AGCCTCACCTATTACCCCTCC | GGGCCACTGAAGAGATGAGAG | SYBR-green assay |
| NR4A3 | AGAGACGTCGTAACAGATGCC | CTCTTTGGTTTGGAAGGCAGC | SYBR-green assay |
| ARC | ACCCAGTTCTTTCCGTGTTTGC | CTGCCCTGCACTTCCCTATC | SYBR-green assay |
| BDNF | GGGTGACAGCAGCAGAGAAA | GACTGGGTAGTTCGGCACTG | FAM-CCAAATGCAACCCCAAGGGG-BHQ1 |
| PGK1 | AAAGTTCAGGATAAGATCCAGCTG | GCCATCAGGTCCTTGACAAT | HEX-GGTGGTGGGATGGCATTCACC-BHQ1 |
| EGR1 | CTGGCTCCCAAGAACTGAAG | AAAGGTTTCTGTCCCGTGTG | HEX-GCGTCCCTATGCCTGCCCAG-BHQ1 |
| HPRT | GTTGGGTTGTGCTGTTTGTG | ATTACCACCCAAAGGTGCAA | FAM-TGCTTAAACCTCATCAACCCAGAACTG-BHQ1 |

**Table S2.** Gene specific **primers. Gene specific** primers for parentage analysis consist of a non-annealing 5’ header sequence and an annealing target-specific sequence. The resulting amplicon incorporates the 5’ header sequences, which support annealing of the index primers in the second PCR.

**Table S3.** **Index primers.** Index primers for parentage analysis contain a 5’ sequence compatible with Illumina sequencing primers (blue), a unique barcode sequence (red), and an annealing sequence (black) specific for the header sequence from the first PCR.

| **Index ID** | **Sequence** |  |  |  |  |
| --- | --- | --- | --- | --- | --- |
| Index_1_F | AATGATACGGCGACCACCGAGATCTACACaaaagggaTCGTCGGCAGCGT | | | | |
| Index_2_F | AATGATACGGCGACCACCGAGATCTACACaaacagccTCGTCGGCAGCGT | | | | |
| Index_3_F | AATGATACGGCGACCACCGAGATCTACACaaactcgcTCGTCGGCAGCGT | | | | |
| Index_4_F | AATGATACGGCGACCACCGAGATCTACACaaagacacTCGTCGGCAGCGT | | | | |
| Index_5_F | AATGATACGGCGACCACCGAGATCTACACtttcctacTCGTCGGCAGCGT | | | | |
| Index_6_F | AATGATACGGCGACCACCGAGATCTACACtggttacaTCGTCGGCAGCGT | | | | |
| Index_7_F | AATGATACGGCGACCACCGAGATCTACACtgcaccatTCGTCGGCAGCGT | | | | |
| Index_8_F | AATGATACGGCGACCACCGAGATCTACACtctgagttTCGTCGGCAGCGT | | | | |
| Index_9_R | CAAGCAGAAGACGGCATACGAGATccatgtgtGTCTCGTGGGCTCGGAGA | | | | |
| Index_10_R | CAAGCAGAAGACGGCATACGAGATgaagacgtGTCTCGTGGGCTCGGAGA | | | | |
| Index_11_R | CAAGCAGAAGACGGCATACGAGATtgtgtggtGTCTCGTGGGCTCGGAGA | | | | |
| Index_12_R | CAAGCAGAAGACGGCATACGAGATgtgtctctGTCTCGTGGGCTCGGAGA | | | | |
| Index_13_R | CAAGCAGAAGACGGCATACGAGATagactgctGTCTCGTGGGCTCGGAGA | | | | |
| Index_14_R | CAAGCAGAAGACGGCATACGAGATtctagcctGTCTCGTGGGCTCGGAGA | | | | |
| Index_15_R | CAAGCAGAAGACGGCATACGAGATcacacgatGTCTCGTGGGCTCGGAGA | | | | |
| Index_16_R | CAAGCAGAAGACGGCATACGAGATgcggtcatGTCTCGTGGGCTCGGAGA | | | | |
| Index_17_R | CAAGCAGAAGACGGCATACGAGATcttcccatGTCTCGTGGGCTCGGAGA | | | | |
| Index_18_R | CAAGCAGAAGACGGCATACGAGATcacctgtgGTCTCGTGGGCTCGGAGA | | | | |
| Index_19_R | CAAGCAGAAGACGGCATACGAGATtagtactgGTCTCGTGGGCTCGGAGA | | | | |
| Index_20_R | CAAGCAGAAGACGGCATACGAGATcaagcatgGTCTCGTGGGCTCGGAGA | | | | |
| Index_21_R | CAAGCAGAAGACGGCATACGAGATttctgtggGTCTCGTGGGCTCGGAGA | | | | |
| Index_22_R | CAAGCAGAAGACGGCATACGAGATagcaagggGTCTCGTGGGCTCGGAGA | | | | |
| Index_23_R | CAAGCAGAAGACGGCATACGAGATgtattaggGTCTCGTGGGCTCGGAGA | | | | |
| Index_24_R | CAAGCAGAAGACGGCATACGAGATaacagtcgGTCTCGTGGGCTCGGAGA | | | | |
| Index_25_R | CAAGCAGAAGACGGCATACGAGATgaacagcgGTCTCGTGGGCTCGGAGA | | | | |
| Index_26_R | CAAGCAGAAGACGGCATACGAGATctgaagcgGTCTCGTGGGCTCGGAGA | | | | |
| Index_27_R | CAAGCAGAAGACGGCATACGAGATaagccgagGTCTCGTGGGCTCGGAGA | | | | |
| Index_28_R | CAAGCAGAAGACGGCATACGAGATacgtgcagGTCTCGTGGGCTCGGAGA | | | | |
| Index_29_R | CAAGCAGAAGACGGCATACGAGATtgagcaagGTCTCGTGGGCTCGGAGA | | | | |
| Index_30_R | CAAGCAGAAGACGGCATACGAGATctgacatcGTCTCGTGGGCTCGGAGA | | | | |
| Index_31_R | CAAGCAGAAGACGGCATACGAGATagggtatcGTCTCGTGGGCTCGGAGA | | | | |
| Index_32_R | CAAGCAGAAGACGGCATACGAGATgttggatcGTCTCGTGGGCTCGGAGA | | | | |
| Index_33_R | CAAGCAGAAGACGGCATACGAGATaacctagcGTCTCGTGGGCTCGGAGA | | | | |
| Index_34_R | CAAGCAGAAGACGGCATACGAGATtgacacgcGTCTCGTGGGCTCGGAGA | | | | |

## Supplementary results

## **IEG expression and playback type**

We tested for directional changes in the expression of individual IEGs across treatment groups, following song playback. We found an interaction between playback type and parental treatment on BDNF expression in CMM. BDNF expression in CMM was lower in response to tutor song in males with two control parents (MCMC.qpcr, tutor playback, Mean=-1.150, P_adj_<0.001), but higher in males with a CORT-treated mother (MCMC.qpcr, tutor playback:CORT mother, Mean=1.372, P_adj_=0.001), a CORT-treated father (MCMC.qpcr, playback:CORT father, Mean=1.145, P_adj_=0.010). or two CORT-treated parents (MCMC.qpcr, playback:CORT mother and father, Mean=1.169, P_adj_=0.010). Males with two CORT-treated parents showed marginally higher CFOS expression (MCMC.qpcr, Mean=0.976, P_adj_=0.057), and there was a marginal increase in CFOS expression following tutor playback (MCMC.qpcr, Mean=0.512, P_adj_=0.080). There was no effect of parental treatment or playback type on IEG expression following song for any other genes examined (P_adj_>0.05 for all). See supplementary materials for the gene-by-gene results.

## **Full IEG expression results**

Table S4. IEG expression in NCM and CMM and parental treatment and playback type. P-values were generated using MCMC.qpcr models and are adjusted for multiple comparisons (false discovery rate). Thinning interval=150, Sample size=2600. N_NCM_=44 and N_CMM_=45 F2 offspring, N=22 parental pairs. M=maternal treatment, F=paternal treatment, PB=playback.

| **Region** | **Gene** | **Factor** | **mode** | **mean** | **lower** | **upper** | **pval.mcmc** | **padj.mcmc** |
| --- | --- | --- | --- | --- | --- | --- | --- | --- |
| CMM | ARC | Control M/CORT F | 0.224 | 0.157 | -0.355 | 0.708 | 0.564 | 0.790 |
| CMM | ARC | CORT M/Control F | 0.052 | 0.108 | -0.376 | 0.621 | 0.675 | 0.843 |
| CMM | ARC | CORT M/CORT F | 0.383 | 0.317 | -0.273 | 0.897 | 0.295 | 0.574 |
| CMM | ARC | PB type (tutor) | 0.374 | 0.326 | -0.169 | 0.739 | 0.156 | 0.364 |
| CMM | ARC | PB type (tutor):Control M/CORT F | -0.099 | 0.007 | -0.617 | 0.624 | 0.981 | 0.981 |
| CMM | ARC | PB type (tutor):CORT M/Control F | -0.179 | -0.205 | -0.850 | 0.432 | 0.535 | 0.790 |
| CMM | ARC | PB type (tutor):CORT M/CORT F | -0.556 | -0.512 | -1.216 | 0.150 | 0.146 | 0.364 |
| CMM | BDNF | Control M/CORT F | -0.605 | -0.547 | -1.015 | -0.007 | 0.037 | 0.117 |
| CMM | BDNF | CORT M/Control F | -0.597 | -0.620 | -1.093 | -0.114 | 0.016 | **0.080** |
| CMM | BDNF | CORT M/CORT F | -0.560 | -0.619 | -1.153 | -0.098 | 0.024 | **0.092** |
| CMM | BDNF | PB type (tutor) | -1.150 | -1.147 | -1.648 | -0.701 | 0.000 | **0.000** |
| CMM | BDNF | PB type (tutor):Control M/CORT F | 1.111 | 1.145 | 0.473 | 1.797 | 0.001 | **0.010** |
| CMM | BDNF | PB type (tutor):CORT M/Control F | 1.368 | 1.372 | 0.703 | 2.072 | 0.000 | **0.001** |
| CMM | BDNF | PB type (tutor):CORT M/CORT F | 1.169 | 1.160 | 0.518 | 1.895 | 0.001 | **0.010** |
| CMM | CFOS | Control M/CORT F | 0.871 | 0.801 | 0.129 | 1.465 | 0.018 | **0.080** |
| CMM | CFOS | CORT M/Control F | 0.699 | 0.617 | 0.046 | 1.214 | 0.040 | 0.118 |
| CMM | CFOS | CORT M/CORT F | 0.976 | 0.963 | 0.281 | 1.665 | 0.008 | **0.057** |
| CMM | CFOS | PB type (tutor) | 0.512 | 0.613 | 0.089 | 1.119 | 0.017 | **0.080** |
| CMM | CFOS | PB type (tutor):Control M/CORT F | -0.056 | -0.042 | -0.730 | 0.660 | 0.908 | 0.950 |
| CMM | CFOS | PB type (tutor):CORT M/Control F | -0.111 | -0.162 | -0.868 | 0.485 | 0.645 | 0.836 |
| CMM | CFOS | PB type (tutor):CORT M/CORT F | -0.792 | -0.829 | -1.589 | -0.096 | 0.031 | 0.107 |
| CMM | EGR1 | Control M/CORT F | -0.147 | -0.114 | -0.532 | 0.356 | 0.618 | 0.832 |
| CMM | EGR1 | CORT M/Control F | 0.143 | 0.138 | -0.292 | 0.597 | 0.545 | 0.790 |
| CMM | EGR1 | CORT M/CORT F | 0.143 | 0.168 | -0.306 | 0.641 | 0.488 | 0.776 |
| CMM | EGR1 | PB type (tutor) | 0.228 | 0.265 | -0.156 | 0.702 | 0.232 | 0.477 |
| CMM | EGR1 | PB type (tutor):Control M/CORT F | 0.047 | 0.058 | -0.573 | 0.599 | 0.847 | 0.950 |
| CMM | EGR1 | PB type (tutor):CORT M/Control F | -0.253 | -0.082 | -0.708 | 0.491 | 0.792 | 0.930 |
| CMM | EGR1 | PB type (tutor):CORT M/CORT F | -0.601 | -0.502 | -1.139 | 0.124 | 0.114 | 0.307 |
| CMM | NR4A3 | Control M/CORT F | 0.083 | -0.062 | -0.498 | 0.443 | 0.797 | 0.930 |
| CMM | NR4A3 | CORT M/Control F | 0.291 | 0.176 | -0.266 | 0.675 | 0.456 | 0.761 |
| CMM | NR4A3 | CORT M/CORT F | 0.223 | 0.233 | -0.230 | 0.753 | 0.352 | 0.649 |
| CMM | NR4A3 | PB type (tutor) | 0.021 | -0.023 | -0.480 | 0.444 | 0.923 | 0.950 |
| CMM | NR4A3 | PB type (tutor):Control M/CORT F | 0.224 | 0.271 | -0.321 | 0.949 | 0.404 | 0.708 |
| CMM | NR4A3 | PB type (tutor):CORT M/Control F | 0.070 | 0.037 | -0.601 | 0.689 | 0.909 | 0.950 |
| CMM | NR4A3 | PB type (tutor):CORT M/CORT F | -0.456 | -0.393 | -1.008 | 0.244 | 0.231 | 0.477 |
| NCM | ARC | Control M/CORT F | -0.143 | -0.251 | -0.875 | 0.338 | 0.412 | 0.800 |
| NCM | ARC | CORT M/Control F | -0.528 | -0.634 | -1.201 | -0.094 | 0.025 | 0.445 |
| NCM | ARC | CORT M/CORT F | -0.464 | -0.499 | -1.177 | 0.134 | 0.138 | 0.800 |
| NCM | ARC | PB type (tutor) | 0.146 | 0.179 | -0.289 | 0.636 | 0.450 | 0.800 |
| NCM | ARC | PB type (tutor):Control M/CORT F | 0.415 | 0.326 | -0.309 | 0.996 | 0.336 | 0.800 |
| NCM | ARC | PB type (tutor):CORT M/Control F | 0.121 | 0.167 | -0.419 | 0.811 | 0.603 | 0.800 |
| NCM | ARC | PB type (tutor):CORT M/CORT F | 0.269 | 0.159 | -0.550 | 0.876 | 0.664 | 0.801 |
| NCM | BDNF | Control M/CORT F | -0.372 | -0.252 | -1.025 | 0.496 | 0.520 | 0.800 |
| NCM | BDNF | CORT M/Control F | -0.248 | -0.259 | -0.957 | 0.476 | 0.487 | 0.800 |
| NCM | BDNF | CORT M/CORT F | -0.529 | -0.594 | -1.392 | 0.235 | 0.165 | 0.800 |
| NCM | BDNF | PB type (tutor) | -0.387 | -0.348 | -0.905 | 0.279 | 0.252 | 0.800 |
| NCM | BDNF | PB type (tutor):Control M/CORT F | 0.040 | -0.059 | -0.904 | 0.844 | 0.893 | 0.919 |
| NCM | BDNF | PB type (tutor):CORT M/Control F | 0.345 | 0.214 | -0.635 | 0.952 | 0.602 | 0.800 |
| NCM | BDNF | PB type (tutor):CORT M/CORT F | 0.588 | 0.518 | -0.336 | 1.394 | 0.252 | 0.800 |
| NCM | CFOS | Control M/CORT F | -0.117 | -0.173 | -0.773 | 0.413 | 0.576 | 0.800 |
| NCM | CFOS | CORT M/Control F | -0.344 | -0.357 | -0.914 | 0.166 | 0.196 | 0.800 |
| NCM | CFOS | CORT M/CORT F | 0.039 | -0.083 | -0.769 | 0.538 | 0.805 | 0.880 |
| NCM | CFOS | PB type (tutor) | 0.148 | 0.120 | -0.353 | 0.584 | 0.623 | 0.800 |
| NCM | CFOS | PB type (tutor):Control M/CORT F | 0.438 | 0.451 | -0.200 | 1.115 | 0.185 | 0.800 |
| NCM | CFOS | PB type (tutor):CORT M/Control F | 0.741 | 0.736 | 0.077 | 1.353 | 0.025 | 0.445 |
| NCM | CFOS | PB type (tutor):CORT M/CORT F | -0.421 | -0.201 | -0.899 | 0.516 | 0.583 | 0.800 |
| NCM | EGR1 | Control M/CORT F | -0.085 | -0.043 | -0.619 | 0.485 | 0.883 | 0.919 |
| NCM | EGR1 | CORT M/Control F | -0.152 | -0.170 | -0.715 | 0.372 | 0.543 | 0.800 |
| NCM | EGR1 | CORT M/CORT F | -0.206 | -0.092 | -0.662 | 0.518 | 0.763 | 0.861 |
| NCM | EGR1 | PB type (tutor) | 0.168 | 0.124 | -0.397 | 0.616 | 0.634 | 0.800 |
| NCM | EGR1 | PB type (tutor):Control M/CORT F | 0.385 | 0.293 | -0.366 | 1.123 | 0.434 | 0.800 |
| NCM | EGR1 | PB type (tutor):CORT M/Control F | 0.353 | 0.327 | -0.387 | 1.038 | 0.362 | 0.800 |
| NCM | EGR1 | PB type (tutor):CORT M/CORT F | 0.188 | 0.213 | -0.579 | 0.960 | 0.590 | 0.800 |
| NCM | NR4A3 | Control M/CORT F | -0.179 | -0.118 | -0.603 | 0.375 | 0.640 | 0.800 |
| NCM | NR4A3 | CORT M/Control F | -0.170 | -0.243 | -0.719 | 0.203 | 0.308 | 0.800 |
| NCM | NR4A3 | CORT M/CORT F | -0.087 | -0.149 | -0.694 | 0.383 | 0.587 | 0.800 |
| NCM | NR4A3 | PB type (tutor) | 0.178 | 0.133 | -0.273 | 0.595 | 0.550 | 0.800 |
| NCM | NR4A3 | PB type (tutor):Control M/CORT F | 0.049 | 0.163 | -0.439 | 0.816 | 0.611 | 0.800 |
| NCM | NR4A3 | PB type (tutor):CORT M/Control F | -0.104 | -0.013 | -0.583 | 0.568 | 0.964 | 0.964 |
| NCM | NR4A3 | PB type (tutor):CORT M/CORT F | 0.080 | 0.129 | -0.530 | 0.802 | 0.705 | 0.822 |

Table S5. IEG expression in NCM and CMM and parental treatment and song similarity between playback song and the target male’s own song. P-values were generated using MCMC.qpcr models and are adjusted for multiple comparisons (false discovery rate). Thinning interval=15, Sample size=2600. N_NCM_=34 and N_CMM_=35 F2 offspring, N=20 parental pairs. M=maternal treatment, F=paternal treatment, PB=playback.

| **Region** | **Gene** | **Factor** | **mode** | **mean** | **lower** | **upper** | **pval.mcmc** | **padj.mcmc** |
| --- | --- | --- | --- | --- | --- | --- | --- | --- |
| CMM | ARC | Control M/CORT F | -1.627 | -1.311 | -3.710 | 1.170 | 0.302 | 0.365 |
| CMM | ARC | CORT M/Control F | -2.953 | -2.910 | -4.909 | -0.913 | 0.005 | **0.050** |
| CMM | ARC | CORT M/CORT F | -1.648 | -1.576 | -4.411 | 1.108 | 0.266 | 0.365 |
| CMM | ARC | PB dissimilarity | -15.004 | -14.856 | -28.426 | -0.798 | 0.037 | 0.143 |
| CMM | ARC | PB dissimilarity:Control M/CORT F | 6.283 | 10.859 | -10.310 | 31.527 | 0.313 | 0.365 |
| CMM | ARC | PB dissimilarity:CORT M/Control F | 18.150 | 22.547 | 6.803 | 39.615 | 0.008 | **0.050** |
| CMM | ARC | PB dissimilarity:CORT M/CORT F | 14.387 | 13.517 | -9.495 | 35.218 | 0.226 | 0.365 |
| CMM | BDNF | Control M/CORT F | 2.916 | 2.183 | -0.482 | 4.761 | 0.109 | 0.295 |
| CMM | BDNF | CORT M/Control F | 3.453 | 2.878 | 0.801 | 5.095 | 0.009 | **0.050** |
| CMM | BDNF | CORT M/CORT F | 1.156 | 1.491 | -1.215 | 4.391 | 0.297 | 0.365 |
| CMM | BDNF | PB dissimilarity | 21.285 | 21.246 | 6.300 | 36.160 | 0.005 | **0.050** |
| CMM | BDNF | PB dissimilarity:Control M/CORT F | -13.824 | -18.113 | -39.646 | 3.791 | 0.109 | 0.295 |
| CMM | BDNF | PB dissimilarity:CORT M/Control F | -24.103 | -24.268 | -41.616 | -5.951 | 0.007 | **0.050** |
| CMM | BDNF | PB dissimilarity:CORT M/CORT F | -14.931 | -13.476 | -35.722 | 8.119 | 0.227 | 0.365 |
| CMM | CFOS | Control M/CORT F | -1.699 | -1.863 | -4.627 | 0.844 | 0.193 | 0.365 |
| CMM | CFOS | CORT M/Control F | -2.869 | -3.092 | -5.550 | -0.722 | 0.014 | **0.060** |
| CMM | CFOS | CORT M/CORT F | -2.014 | -1.775 | -4.823 | 1.395 | 0.266 | 0.365 |
| CMM | CFOS | PB dissimilarity | -18.525 | -20.120 | -35.335 | -4.319 | 0.012 | **0.060** |
| CMM | CFOS | PB dissimilarity:Control M/CORT F | 22.395 | 18.662 | -3.720 | 42.189 | 0.119 | 0.298 |
| CMM | CFOS | PB dissimilarity:CORT M/Control F | 26.135 | 27.295 | 7.823 | 45.403 | 0.005 | **0.050** |
| CMM | CFOS | PB dissimilarity:CORT M/CORT F | 18.506 | 18.190 | -5.956 | 41.799 | 0.139 | 0.325 |
| CMM | EGR1 | Control M/CORT F | -1.763 | -1.564 | -3.956 | 0.645 | 0.187 | 0.365 |
| CMM | EGR1 | CORT M/Control F | -0.997 | -1.175 | -2.948 | 0.740 | 0.214 | 0.365 |
| CMM | EGR1 | CORT M/CORT F | -2.325 | -2.357 | -4.665 | 0.023 | 0.053 | 0.186 |
| CMM | EGR1 | PB dissimilarity | -5.871 | -7.517 | -21.054 | 4.937 | 0.248 | 0.365 |
| CMM | EGR1 | PB dissimilarity:Control M/CORT F | 15.583 | 11.741 | -7.686 | 30.478 | 0.230 | 0.365 |
| CMM | EGR1 | PB dissimilarity:CORT M/Control F | 8.144 | 9.656 | -5.420 | 25.168 | 0.217 | 0.365 |
| CMM | EGR1 | PB dissimilarity:CORT M/CORT F | 18.488 | 17.992 | -0.061 | 37.196 | 0.060 | 0.191 |
| CMM | NR4A3 | Control M/CORT F | -0.221 | -0.389 | -2.799 | 2.040 | 0.754 | 0.776 |
| CMM | NR4A3 | CORT M/Control F | -0.502 | -0.583 | -2.440 | 1.478 | 0.561 | 0.613 |
| CMM | NR4A3 | CORT M/CORT F | -1.337 | -1.351 | -3.906 | 0.924 | 0.286 | 0.365 |
| CMM | NR4A3 | PB dissimilarity | -0.195 | -1.381 | -15.738 | 11.515 | 0.844 | 0.844 |
| CMM | NR4A3 | PB dissimilarity:Control M/CORT F | 2.668 | 3.661 | -17.158 | 22.290 | 0.721 | 0.765 |
| CMM | NR4A3 | PB dissimilarity:CORT M/Control F | 4.404 | 6.264 | -11.240 | 20.941 | 0.450 | 0.508 |
| CMM | NR4A3 | PB dissimilarity:CORT M/CORT F | 11.874 | 10.818 | -8.020 | 30.436 | 0.278 | 0.365 |
| NCM | ARC | Control M/CORT F | -0.784 | -0.803 | -3.716 | 2.291 | 0.595 | 0.993 |
| NCM | ARC | CORT M/Control F | -2.798 | -2.745 | -4.951 | -0.697 | 0.010 | 0.115 |
| NCM | ARC | CORT M/CORT F | 0.241 | 0.184 | -2.715 | 3.226 | 0.904 | 0.993 |
| NCM | ARC | PB dissimilarity | -24.384 | -23.542 | -37.096 | -9.158 | 0.001 | **0.035** |
| NCM | ARC | PB dissimilarity:Control M/CORT F | 4.949 | 3.439 | -23.948 | 29.359 | 0.797 | 0.993 |
| NCM | ARC | PB dissimilarity:CORT M/Control F | 16.176 | 17.800 | 1.367 | 34.630 | 0.035 | 0.209 |
| NCM | ARC | PB dissimilarity:CORT M/CORT F | -8.973 | -2.195 | -26.492 | 20.352 | 0.855 | 0.993 |
| NCM | BDNF | Control M/CORT F | 0.428 | 0.323 | -3.395 | 4.011 | 0.866 | 0.993 |
| NCM | BDNF | CORT M/Control F | 0.776 | 0.712 | -1.996 | 3.194 | 0.587 | 0.993 |
| NCM | BDNF | CORT M/CORT F | -1.242 | -2.332 | -5.944 | 1.510 | 0.223 | 0.879 |
| NCM | BDNF | PB dissimilarity | -0.274 | 2.109 | -14.971 | 19.360 | 0.808 | 0.993 |
| NCM | BDNF | PB dissimilarity:Control M/CORT F | -0.911 | -1.380 | -35.662 | 30.876 | 0.936 | 0.993 |
| NCM | BDNF | PB dissimilarity:CORT M/Control F | -9.754 | -5.388 | -27.165 | 14.170 | 0.606 | 0.993 |
| NCM | BDNF | PB dissimilarity:CORT M/CORT F | 11.831 | 16.137 | -13.492 | 43.820 | 0.280 | 0.879 |
| NCM | CFOS | Control M/CORT F | 0.222 | -0.235 | -3.082 | 3.099 | 0.883 | 0.993 |
| NCM | CFOS | CORT M/Control F | -1.050 | -1.205 | -3.249 | 1.015 | 0.276 | 0.879 |
| NCM | CFOS | CORT M/CORT F | -1.119 | -0.874 | -4.113 | 2.221 | 0.591 | 0.993 |
| NCM | CFOS | PB dissimilarity | -14.255 | -14.129 | -28.333 | 1.087 | 0.060 | 0.302 |
| NCM | CFOS | PB dissimilarity:Control M/CORT F | -7.387 | -0.724 | -28.019 | 26.998 | 0.959 | 0.993 |
| NCM | CFOS | PB dissimilarity:CORT M/Control F | 8.481 | 9.608 | -7.513 | 26.767 | 0.278 | 0.879 |
| NCM | CFOS | PB dissimilarity:CORT M/CORT F | 4.054 | 7.561 | -17.830 | 32.065 | 0.551 | 0.993 |
| NCM | EGR1 | Control M/CORT F | -0.452 | -0.479 | -3.667 | 2.665 | 0.763 | 0.993 |
| NCM | EGR1 | CORT M/Control F | 0.416 | -0.009 | -2.144 | 2.283 | 0.993 | 0.993 |
| NCM | EGR1 | CORT M/CORT F | 0.396 | 0.710 | -2.465 | 3.952 | 0.659 | 0.993 |
| NCM | EGR1 | PB dissimilarity | -3.872 | -5.137 | -20.820 | 10.011 | 0.513 | 0.993 |
| NCM | EGR1 | PB dissimilarity:Control M/CORT F | 4.592 | 4.204 | -22.129 | 31.807 | 0.758 | 0.993 |
| NCM | EGR1 | PB dissimilarity:CORT M/Control F | 0.667 | -0.092 | -18.767 | 16.788 | 0.992 | 0.993 |
| NCM | EGR1 | PB dissimilarity:CORT M/CORT F | -3.358 | -5.130 | -30.754 | 18.993 | 0.685 | 0.993 |
| NCM | NR4A3 | Control M/CORT F | -0.937 | -0.712 | -3.284 | 2.037 | 0.608 | 0.993 |
| NCM | NR4A3 | CORT M/Control F | -2.345 | -2.224 | -4.242 | -0.329 | 0.025 | 0.209 |
| NCM | NR4A3 | CORT M/CORT F | -0.570 | -1.326 | -4.201 | 1.441 | 0.359 | 0.968 |
| NCM | NR4A3 | PB dissimilarity | -18.894 | -19.404 | -32.605 | -5.783 | 0.005 | **0.084** |
| NCM | NR4A3 | PB dissimilarity:Control M/CORT F | 2.588 | 4.568 | -19.138 | 27.135 | 0.704 | 0.993 |
| NCM | NR4A3 | PB dissimilarity:CORT M/Control F | 17.931 | 16.738 | 0.780 | 32.436 | 0.036 | 0.209 |
| NCM | NR4A3 | PB dissimilarity:CORT M/CORT F | 16.746 | 11.649 | -10.693 | 33.004 | 0.301 | 0.879 |

Table S6 Estimates of extra-pair parentage (EPP) based on paternity analyses using the COLONY package. To account for potential sequencing errors, we estimated the closest match for each father by allowing for two mismatches. Males that matched closest with an F1 male that was not their social father (confirmed EPP) are highlighted in bold font, as are F2 males that were not assigned to any potential father in the dataset (unconfirmed). The extra-pair paternity rates are displayed at the bottom of the table.

| F2 male ID | Paternal treatment | Social father | Best fit | Pairwise confidence |
| --- | --- | --- | --- | --- |
| O119 | Control | O180 | O180 | 100% |
| O120 | Control | O180 | O180 | 100% |
| O193 | Control | O180 | O180 | 100% |
| O198 | CORT | O037 | O037 | 100% |
| O335 | Control | O180 | **O037** | **55%** |
| O340 | Control | O180 | O180 | 100% |
| O353 | Control | O180 | O180 | 100% |
| O367 | CORT | O037 | O037 | 100% |
| O369 | CORT | O037 | **not assigned** |  |
| O375 | CORT | O272 | O272 | 100% |
| O376 | CORT | O272 | O272 | 100% |
| O387 | Control | O017 | O017 | 100% |
| O389 | Control | O017 | O017 | 100% |
| O394 | Control | O180 | O180 | 100% |
| O396 | Control | O180 | O180 | 100% |
| O399 | Control | O188 | O188 | 100% |
| O403 | Control | O057 | O057 | 100% |
| O407 | Control | O057 | O057 | 100% |
| O411 | CORT | O048 | O048 | 100% |
| O413 | CORT | O048 | O048 | 100% |
| O418 | Control | O057 | O057 | 100% |
| O419 | Control | O057 | O057 | 100% |
| O425 | Control | O190 | O190 | 100% |
| O426 | Control | O190 | **O272** | **55%** |
| O427 | Control | O236 | O236 | 100% |
| O438 | Control | O236 | O236 | 100% |
| O439 | CORT | O058 | **not assigned** |  |
| O442 | CORT | O058 | **not assigned** |  |
| O451 | CORT | O037 | **O272** | **50%** |
| O463 | CORT | O037 | O037 | 100% |
| O464 | CORT | O037 | O037 | 100% |
| O465 | CORT | O037 | O037 | 100% |
| O466 | CORT | O075 | O075 | 100% |
| O467 | CORT | O075 | O075 | 100% |
| O475 | CORT | O075 | O075 | 100% |
| O477 | CORT | O075 | O075 | 100% |
| O482 | Control | O180 | O180 | 100% |
| O483 | Control | O180 | O180 | 100% |
| O488 | Control | O224 | O224 | 100% |
| O489 | Control | O224 | O224 | 100% |
| O491 | Control | O188 | O188 | 100% |
| O502 | CORT | O272 | O272 | 100% |
| Extra-pair paternity | | Confirmed | | 8% (3/39) |
|  |  | Unassigned included | | 14% (6/42) |

Table S7 Estimates of extra-pair parentage (EPP) based on maternity analyses using the COLONY package. To account for potential sequencing errors, we estimated the closest match for each mother by allowing for two mismatches. Males that were not assigned to any potential mother in the dataset (unconfirmed) are highlighted in bold font. The extra-pair maternity rates are displayed at the bottom of the table.

| F2 male ID | Maternal treatment | Social mother | Best fit | Pairwise confidence |
| --- | --- | --- | --- | --- |
| O056 | Control | O270 | O270 | 100% |
| O073 | Control | O039 | O039 | 100% |
| O085 | Control | O039 | O039 | 100% |
| O198 | Control | O268 | O268 | 100% |
| O214 | Control | O039 | O039 | 100% |
| O335 | Control | O200 | O200 | 100% |
| O353 | Control | O200 | O200 | 100% |
| O363 | CORT | O184 | O184 | 100% |
| O367 | Control | O268 | O268 | 100% |
| O369 | Control | O268 | O268 | 100% |
| O375 | Control | O200 | O200 | 100% |
| O376 | Control | O200 | O200 | 100% |
| O387 | CORT | O199 | O199 | 100% |
| O389 | CORT | O199 | O199 | 100% |
| O392 | Control | O039 | O039 | 100% |
| O394 | Control | O200 | O200 | 100% |
| O396 | Control | O200 | O200 | 100% |
| O399 | Control | O249 | O249 | 100% |
| O411 | Control | O273 | O273 | 100% |
| O413 | Control | O273 | O273 | 100% |
| O418 | Control | O082 | O082 | 100% |
| O419 | Control | O082 | O082 | 100% |
| O425 | CORT | O184 | O184 | 100% |
| O426 | CORT | O184 | O184 | 100% |
| O427 | CORT | O032 | **not assigned** |  |
| O439 | CORT | O211 | O211 | 100% |
| O442 | CORT | O211 | O211 | 100% |
| O451 | CORT | O211 | O211 | 100% |
| O463 | CORT | O211 | O211 | 100% |
| O464 | CORT | O211 | **not assigned** |  |
| O465 | CORT | O211 | O211 | 100% |
| O466 | Control | O082 | O082 | 100% |
| O467 | Control | O082 | O082 | 100% |
| O482 | CORT | O061 | O061 | 100% |
| O483 | CORT | O061 | O061 | 100% |
| O488 | CORT | O199 | O199 | 100% |
| O489 | CORT | O199 | O199 | 100% |
| O491 | Control | O249 | O249 | 100% |
| O502 | CORT | O210 | O210 | 100% |
| Extra-pair maternity | | Confirmed | | 0% (0/37) |
|  |  | Unassigned included | | 5% (2/39) |

## **Song similarity within breeding experiment rooms**

We plotted song similarity in the two breeding aviaries as Euclidean coordinates in two dimensions (Fig. S2). We did not see a close association between social fathers and offspring overall, and the similarity between social fathers and offspring varied between social fathers (Fig. S2).


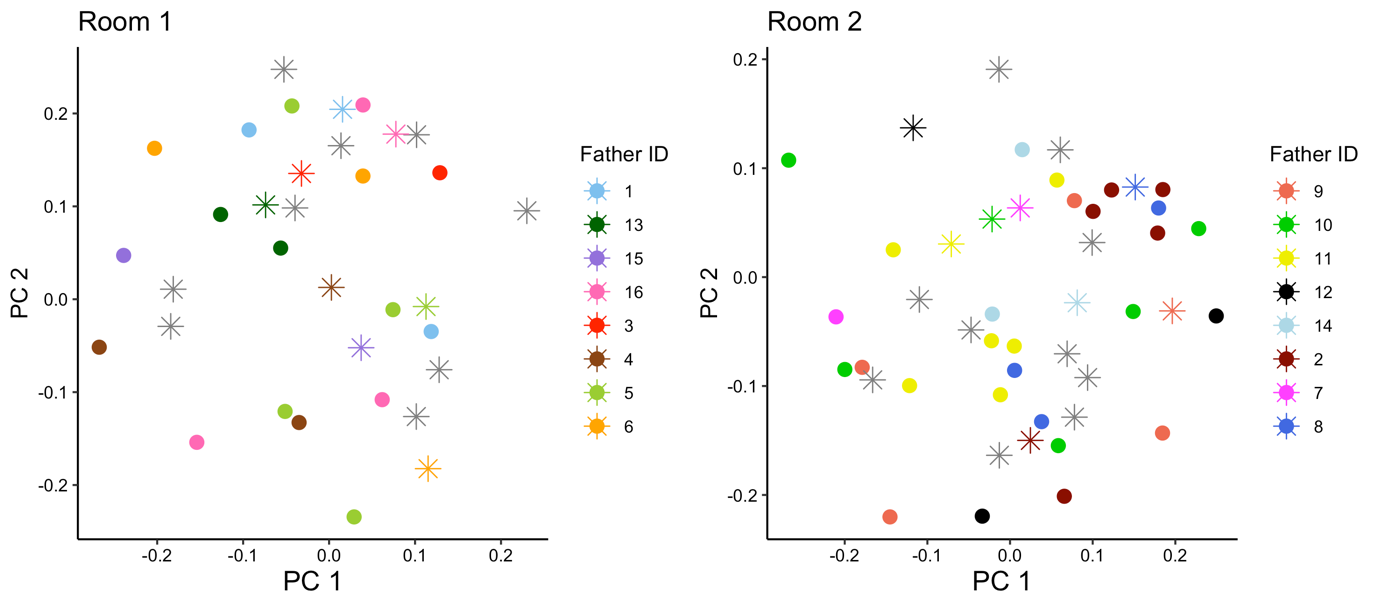


Figure S2. The song similarity between F2 males and their social fathers in the two rooms used during the breeding experiment, visualised with Euclidean distance (generated using Luscinia nonmetric MDS). Points closer together represent individuals whose songs are more acoustically similar. Colours represent social fathers (stars) and their offspring (points). Grey stars represent F1 males that did not sire offspring that were included in this study. While the songs of some F2 males closely resembled those of their social father (e.g. social father 13 in room 1, dark green), others did not closely resemble their social father (e.g. social father 12 in room 2, black). Interestingly, some F2 male siblings produced songs that closely resembled the songs of an F1 male that was not their social father (see dark red offspring of father 2 being closely associated with blue father 8 in room 2), suggesting that these males may have learned their songs from this male rather than their social father.

**Father tutor rank**

We did not find any statistical evidence that the tutor rank differed between treatment groups (*X*-squared = 3.7675, df = 3, p-value = 0.288, table S8). However, observations of the rank distribution indicated that males with two control parents had more top-ranked fathers, while birds with two CORT-treated parents had more bottom-ranked social fathers (Fig. S3).

Table S8. Contingency table of the observed and expected number of father tutor ranks for each father/son comparison in the tutor rank quartiles. The expected values are based on the observed overall distribution of tutor ranks across all treatment groups. The expected numbers are higher in the CORT/CORT group because there were slightly more males in this treatment group. While F2 males with one CORT-treated parent had observed values similar to expected values (yellow and green), birds with two control parents had slightly more top-ranked social fathers (blue), and birds with two CORT-treated parents had slightly more bottom-ranked social fathers (red). The differences between treatment groups were not statistically significant (P=0.288).

|  | Father tutor rank | | | |
| --- | --- | --- | --- | --- |
| Parental treatment | Top ranked  (High song similarity) | | Bottom ranked  (Low song similarity) | |
|  | Expected | Observed | Expected | Observed |
| Control ♂ / Control ♀  N=11 | 5.62 | 8 | 5.38 | 3 |
| Control ♂ / CORT ♀  N=11 | 5.62 | 6 | 5.38 | 5 |
| CORT ♂ / Control ♀  N=11 | 5.62 | 5 | 5.38 | 6 |
| CORT ♂ / CORT ♀  N=12 | 6.13 | 4 | 5.87 | 8 |


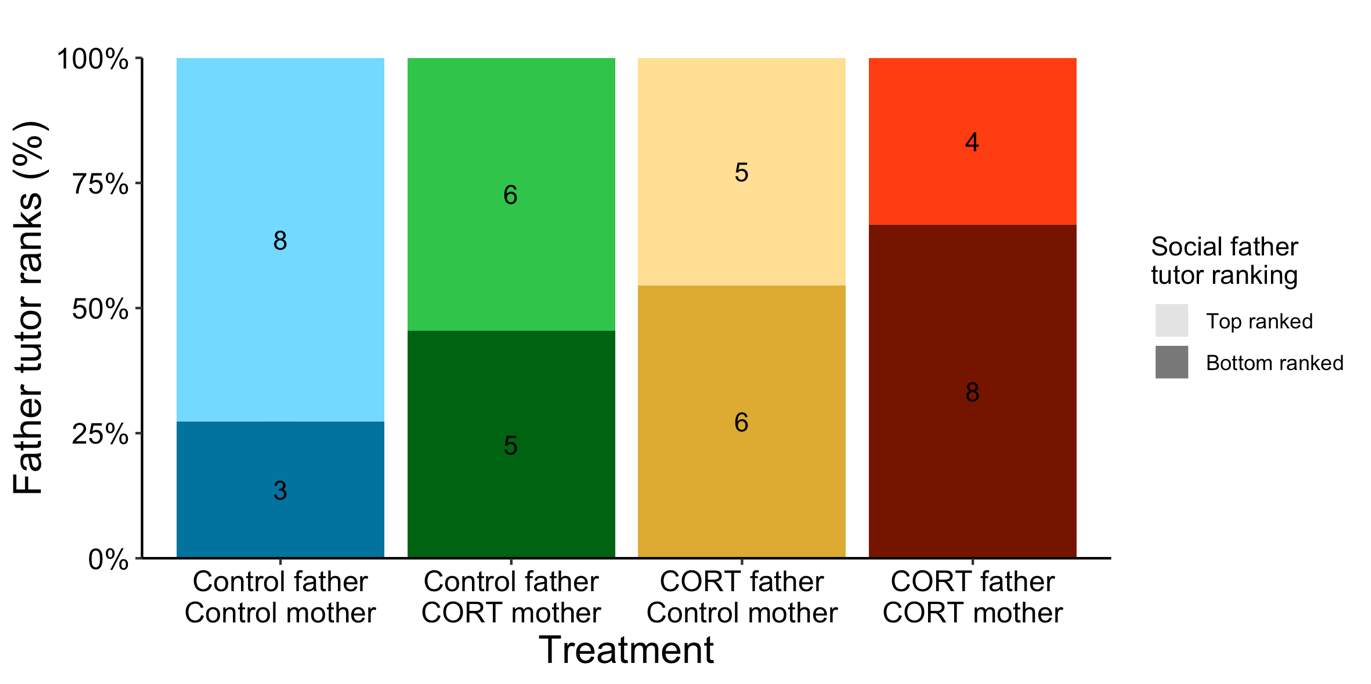


Figure S3. The distribution of the social father’s tutor rank across parental treatment groups of all comparisons between the social father’s song and the son’s song. The numbers displayed over the bars represent the number of F2 males whose fathers ranked in the top or bottom 50% of all potential tutors. For example, for the treatment group with two control parents, eight F2 male’s social fathers ranked in the top 50% of potential tutors, and three F2 male’s social fathers ranked in the bottom 50% of potential tutors. Note that there was one more male in the treatment group with two CORT-treated parents (red). N=45 F2 males

***References***

Boogert, N. J., Lachlan, R. F., Spencer, K. A., Templeton, C. N., & Farine, D. R. (2018). Stress hormones, social associations and song learning in zebra finches. *Philos. Trans. R. Soc. B*, *373*(1756), 1-9. <https://doi.org/10.1098/rstb.2017.0290>

Clayton, D. F., Anreiter, I., Aristizabal, M., Frankland, P. W., Binder, E. B., & Citri, A. (2019). The role of the genome in experience-dependent plasticity: Extending the analogy of the genomic action potential. *PNAS*, 201820837. <https://doi.org/10.1073/pnas.1820837116> %J Proceedings of the National Academy of Sciences

Crino, O. L., Jensen, S. M., Buchanan, K. L., & Griffith, S. C. (2018). Evidence for condition mediated trade-offs between the HPA- and HPG-axes in the wild zebra finch. *Gen. Comp. Endocrinol.*, *259*, 189-198. <https://doi.org/https://doi.org/10.1016/j.ygcen.2017.11.025>

Dawson, D. A., Horsburgh, G. J., Küpper, C., Stewart, I. R., Ball, A. D., Durrant, K. L., . . . Burke, T. (2010). New methods to identify conserved microsatellite loci and develop primer sets of high cross-species utility - as demonstrated for birds. *Mol Ecol Resour*, *10*(3), 475-494. <https://doi.org/10.1111/j.1755-0998.2009.02775.x>

Kraft, F.-L. O. H., Driscoll, S. C., Buchanan, K. L., & Crino, O. L. (2019). Developmental stress reduces body condition across avian life-history stages: A comparison of quantitative magnetic resonance data and condition indices. *Gen. Comp. Endocrinol.*, *272*, 33-41. <https://doi.org/https://doi.org/10.1016/j.ygcen.2018.11.008>

Kraft, F. H., Crino, O. L., & Buchanan, K. L. (2021). Developmental conditions have intergenerational effects on corticosterone levels in a passerine. *Horm Behav*, *134*, 105023. <https://doi.org/10.1016/j.yhbeh.2021.105023>

Mello, C. V., & Clayton, D. F. (1994). Song-induced ZENK gene expression in auditory pathways of songbird brain and its relation to the song control system. *J Neurosci*, *14*(11 Pt 1), 6652-6666. <https://doi.org/10.1523/JNEUROSCI.14-11-06652.1994>

Mello, C. V., Vicario, D. S., & Clayton, D. F. (1992). Song presentation induces gene expression in the songbird forebrain. *PNAS*, *89*, 6818-6822.

Nag, S., Dalgaard, M. D., Kofoed, P. E., Ursing, J., Crespo, M., Andersen, L. O., . . . Alifrangis, M. (2017). High throughput resistance profiling of Plasmodium falciparum infections based on custom dual indexing and Illumina next generation sequencing-technology. *Sci Rep*, *7*(1), 2398. <https://doi.org/10.1038/s41598-017-02724-x>

Qamar, W., Khan, M. R., & Arafah, A. (2017). Optimization of conditions to extract high quality DNA for PCR analysis from whole blood using SDS-proteinase K method. *Saudi J Biol Sci*, *24*(7), 1465-1469. <https://doi.org/10.1016/j.sjbs.2016.09.016>

Spencer, K. A., & Verhulst, S. (2007). Delayed behavioral effects of postnatal exposure to corticosterone in the zebra finch (Taeniopygia guttata). *Horm. Behav.*, *51*(2), 273-280. <https://doi.org/10.1016/j.yhbeh.2006.11.001>

Warren, W. C., Clayton, D. F., Ellegren, H., Arnold, A. P., Hillier, L. W., Künstner, A., . . . Wilson, R. K. (2010). The genome of a songbird. *Nature*, *464*, 757-762. <https://doi.org/10.1038/nature08819>

<https://www.nature.com/articles/nature08819#supplementary-information>
